# Sequencing of Lynch syndrome tumors reveals the importance of epigenetic alterations

**DOI:** 10.18632/oncotarget.22445

**Published:** 2017-11-14

**Authors:** Noora Porkka, Satu Valo, Taina T. Nieminen, Alisa Olkinuora, Satu Mäki-Nevala, Samuli Eldfors, Päivi Peltomäki

**Affiliations:** ^1^ Department of Medical and Clinical Genetics, University of Helsinki, Helsinki, Finland; ^2^ Institute for Molecular Medicine Finland, University of Helsinki, Helsinki, Finland

**Keywords:** Lynch syndrome, colorectal tumor, ovarian cancer, epigenetic regulation, somatic mutation

## Abstract

Genomic instability and epigenetic aberrations are important classifiers of human tumors, yet, their interrelations are poorly understood. We used Lynch syndrome (LS) to address such relationships. Forty-five tumors (11 colorectal adenomas, 18 colorectal carcinomas, and 16 ovarian carcinomas) were profiled for CpG Island Methylator Phenotype (CIMP) and somatic mutations. All tumors showed high-degree microsatellite instability. Panel sequencing of 578 cancer-relevant genes revealed the average number of 1433, 1124, and 657 non-synonymous somatic mutations per colorectal adenoma, colorectal carcinoma, and ovarian carcinoma, respectively. Genes harboring mutations with allele frequency 25 % or higher in at least 31 % of tumors were regarded to be possible drivers. Among 72 and 10 such genes identified in colorectal and ovarian tumors, respectively, the most frequently mutated genes *BRD4* and *MLL2* (62 % of colorectal tumors) and *ARID1A* (50 % of ovarian carcinomas) are involved in epigenetic regulation. The total number of somatic mutations or mutant genes per tumor were significantly associated with CIMP. Our results suggest that even in an inherited disease, tumor type-specific epigenetic changes are significant and may result from regulatory changes (CIMP) or structural events (mutations of epigenetic regulatory genes). The findings are clinically relevant since many of the affected pathways can be therapeutically targeted.

## INTRODUCTION

Genomic instability and epigenetic aberrations divide human tumors into biologically and clinically meaningful subgroups. Large-scale sequencing studies show that 16 % of colorectal carcinomas are hypermutated and most of these exhibit high-degree microsatellite instability (MSI-H) [1]. In sporadic tumors, biallelic *MLH1* promoter methylation as part of a generalized CpG island methylator phenotype (CIMP) is the main mechanism behind MSI-H. Similarly, genomic and epigenomic profiling classifies epithelial ovarian carcinomas [2] and endometrial carcinomas [3] into separate subgroups with distinct behavior and different requirements for clinical management. Current evidence supports the existence of pan-cancer CIMP [4]. However, a universal, causal set of mutations driving CIMP is yet to be identified.

Lynch syndrome (LS) is a well-established cancer predisposition syndrome with an increased risk of colorectal, endometrial, ovarian, and other malignancies resulting from germline mutations in DNA mismatch repair (MMR) genes *MLH1*, *MSH2*, *MSH6*, and *PMS2* [5]. Contrary to sporadic MSI-H tumors, two-hit inactivation in microsatellite-unstable LS tumors occurs by a genetic mechanism, typically loss of heterozygosity (LOH) [6]. The patterns of CIMP in tumor tissues from LS patients overlap with those of the corresponding sporadic tumors [6]. LS tumors may also exhibit distinct profiles of somatic mutations and epigenetic aberrations; for example, we have described a unique molecular profile for ovarian carcinomas in LS, possibly explaining the surprisingly favorable outcome of LS-associated disease [7].

Our recent investigation showed that DNA hypermethylation accompanies increasing dysplasia in LS-associated colorectal tumorigenesis [8]. To gain insight into the interplay between genetic and epigenetic events, which is poorly understood at present, we now set out to determine the somatic mutation profiles of 29 colorectal tumors from the same series by targeted sequencing. To address the question of tumor-type specificity of acquired molecular changes in this multiorgan cancer syndrome where the predisposing mutation is present in every cell, 16 ovarian carcinomas from LS mutation carriers were examined for comparison. Our findings reveal organ-specific patterns of mutant genes and a close connection between hypermutability and CIMP.

## RESULTS

### Mechanisms of MMR gene inactivation

Among 11 colorectal adenomas, 18 colorectal carcinomas, and 16 ovarian carcinomas examined from MMR gene mutation carriers, all showed high-degree MSI (Table 1). The first (germline) and second (somatic) hits are shown case by case in Supplementary Table 1 and summarized in Table 2. LOH, somatic point mutations, and promoter methylation were addressed as possible second hits. Loss of the wild-type allele of the MMR gene involved was the most common second hit, being particularly frequent in colorectal adenomas (9/11, 82 %), but also in those colorectal carcinomas (9/17, 53 %) and ovarian carcinomas (7/11, 64 %) that could be assessed for second hits. Somatic point mutations in the predisposing MMR genes provided obvious second hits for most cases without LOH. *MLH1* was our particular focus in methylation analyses; no increased methylation in tumor tissue was detected in any *MLH1*-associated cases.

**Table 1:** Characteristics of LS sample series

	Predisposing gene			MMR status		CIMP status^*^		
	*MLH1*	*MSH2*	*MSH6*	MSI (high)	MSS	Negative	Positive	ND
**Colorectal specimens (n = 29)**	**22 (76 %)**	**3 (10 %)**	**4 (14 %)**	**29 (100 %)**	**0**	**18 (62 %)**	**11 (38 %)**	**0**
Adenoma (n = 11)	10 (91 %)	1 (9 %)	0	11 (100 %)	0	9 (82 %)	2 (18 %)	0
Carcinoma (n = 18)	12 (67 %)	2 (11 %)	4 (22 %)	18 (100 %)	0	9 (50 %)	9 (50 %)	0
**Ovarian carcinoma (n = 16)**	**13 (81 %)**	**3 (19 %)**	**0**	**16 (100 %)**	**0**	**12 (75 %)**	**3 (19 %)**	**1 (6 %)**
**Total (n = 45)**	**35 (78 %9**	**6 (13 %)**	**4 (9 %)**	**45 (100 %)**	**0**	**30 (67 %)**	**14 (31 %)**	**1 (2 %)**

^*^Determined by MS-MLPA (ME042-B2 for colorectal tumors and ME001-C1 for ovarian carcinomas; see Materials and Methods).

ND, could not be determined due to shortage of DNA.

**Table 2:** Mechanisms of two-hit inactivation of MMR genes in LS tumors

	Germline mutation + LOH	Germline mutation + somatic point mutation	No obvious second hit	ND
**Colorectal adenoma (n = 11)**	9	2	0	0
**Colorectal carcinoma (n = 18)**	9	6	2	1
**Ovarian carcinoma (n = 16)**	7	3	1	5

Mutations that had the possibility of being pathogenic (pathogenic, likely pathogenic, or pathogenicity unknown) were considered (please see Supplementary Table 1 for pathogenicity class for each mutation).

ND, not determined.

### Profiles of somatic mutations stratified by CIMP

The average number of nonsynonymous somatic mutations was 1442, 1124, and 657 per colorectal adenoma, colorectal carcinoma, and ovarian carcinoma, respectively (Table 3). All colorectal adenomas had high-grade dysplasia and were combined with colorectal carcinomas in further analyses. The mean number of somatic mutations in colorectal tumors (1245) was significantly higher compared to ovarian carcinomas (P = 0.0004). The average number of mutant genes out of 578 examined is given in two ways in Table 3, using a mutant allele frequency of 25 % as a divider between high and low-frequency mutations [9]. The mean number of genes with nonsynonymous high-frequency mutations was 2.9-, 1.5-, and 2.3-fold higher in CIMP positive than CIMP negative colorectal adenomas colorectal carcinomas, and ovarian carcinomas, respectively (in ovarian carcinomas, the difference was evident even without restriction to high-frequency mutations). When colorectal and ovarian tumors were combined, the difference between CIMP positive and CIMP negative tumors was statistically significant irrespective of the somatic mutation parameter (number of somatic mutations or mutant genes) used (Table 3). To demonstrate that the result did not depend on any specific system of CIMP classification, we additionally examined our colorectal tumors with the same MS-MLPA assay used for ovarian tumors (see Materials and Methods) and performed correlation analysis on the combined set of tumors by treating methylation status as a continuous variable (Supplementary Table 2). The number of methylated genes out of 24 showed a significant positive correlation with the number of mutant genes in the same tumors (*p* = 0.002 considering any mutant allele frequency and *p* = 0.006 if only genes affected with high-frequency mutations were taken into account). Collectively, our results indicated that CIMP status significantly influenced the tendency to acquire somatic mutations.

**Table 3:** Average numbers of somatic non-synonymous mutations and mutant genes among 578 cancer-relevant genes investigated

	Average no. of non-synonymous mutations	*p*-value	Average no. of mutant genes (any frequency for mutant alleles)	*p*-value	Average no. of mutant genes (frequency ≥ 25 % for mutant alleles)	*p*-value
**Colorectal adenoma (n = 11)**	**1442**		**370**		**78**	
CIMP-negative (n = 9)	1433	ns	355	ns	58	ns (0.099)
CIMP-positive (n = 2)	1485		440		170	
**Colorectal carcinoma (n = 18)**	**1124**		**361**		**97**	
CIMP-negative (n = 9)	1050	ns	346	ns	77	ns
CIMP-positive (n = 9)	1198		375		118	
**Colorectal tumors combined (n = 29)**	**1245**		**364**		**90**	
CIMP-negative (n = 18)	1242	ns	351	ns	67	ns (0.076)
CIMP-positive (n = 11)	1250		387		128	
Ovarian carcinoma (n = 16)^*^	**657**		**162**		**30**	
CIMP-negative (n = 12)	252	0.014	99	0.014	25	0.030
CIMP-positive (n = 3)	2494		464		58	
Colorectal and ovarian tumors combined (n = 45) ^*^	**954**		**287**		**70**	
CIMP-negative (n = 30)	846	0.011	250	0.019	50	0.004
CIMP-positive (n = 14)	1517		403		113	

^*^Includes one ovarian carcinoma with CIMP status unknown.

*p*-values were calculated by Mann-Whitney U test (ns, non-significant).

Case-by-case data on CIMP status vs. no. of mutations or mutant genes are shown in Supplementary Table 2.

### Genes characteristic of colorectal vs. ovarian tumorigenesis

**Figure 1:**
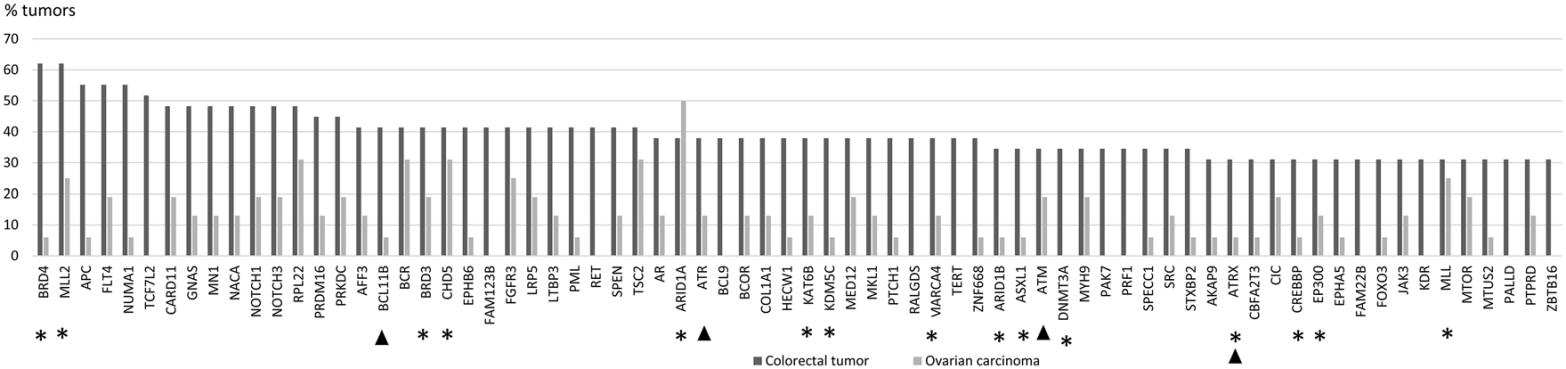
Colorectal tumor-associated genes Genes affected with high-frequency mutations (mutant allele frequency > 25 %) in at least 31 % (9/29) of LS-colorectal tumors are shown. Mutation percentages of the same 72 genes in LS-ovarian carcinomas are displayed for comparison. Epigenetic regulatory genes are marked with an asterisk and DNA repair genes with an arrowhead (please see Supplementary Table 4 for functional annotation of the remaining genes).

**Figure 2:**
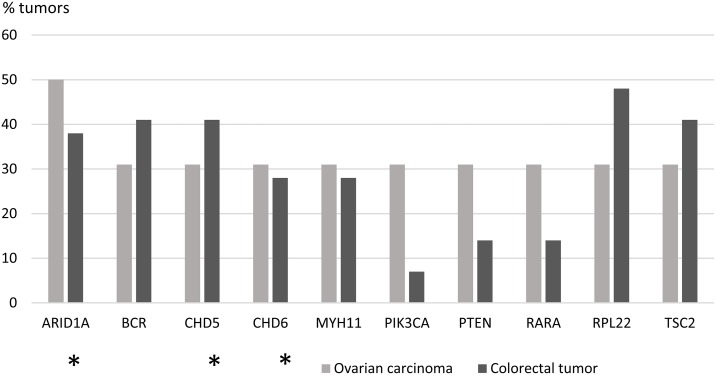
Ovarian carcinoma-associated genes Genes affected with high-frequency mutations (mutant allele frequency > 25 %) in at least 31 % (5/16) of LS-ovarian carcinomas are shown. Mutation percentages of the same 10 genes in LS-colorectal tumors are displayed for comparison. Epigenetic regulatory genes are marked with an asterisk.

The proportion of tumors in which a particular gene was mutant was calculated for each of the 578 genes (Supplementary Table 3). Focusing on mutations with high allele frequencies (> 25 %), 51 genes showed no mutations in colorectal tumors, whereas the other extreme of the distribution consisted of 2 genes mutant in 18 tumors. For ovarian tumors, the distribution of mutant genes ranged from 285 genes showing no high-frequency mutations to 1 gene mutant in 8 tumors. The distribution of mutated genes in colorectal tumors suggested that 9/29 mutant tumors (31 %) provided a cut-off that divided the tumors into commonly and less commonly mutated ones. The same threshold (5/16, 31 %) was subsequently applied to ovarian carcinomas to allow for tumor type-specific comparisons. Using the tumor proportion of 31 % and mutant allele frequency of 25 % or higher as requirements, 72 genes characteristic of colorectal tumorigenesis (Figure 1) and 10 genes characteristic of ovarian tumorigenesis (Figure 2) were identified. Pathway annotation of each gene is given in Supplementary Table 4. Twenty-nine of the 72 colon tumor-associated genes including, e.g., the Wnt-signaling-pathway genes *APC*, *TCF7L2*, and *FAM123B* showed significant specificity for colorectal tumors (Figure 1 and Supplementary Table 4). Likewise, a majority of the 10 ovarian carcinoma-associated genes were preferentially affected in ovarian carcinoma (Figure 2), although the differences did not reach statistical significance (borderline significant difference for *PIK3CA*, Supplementary Table 4). The *ARID1A*, *BCR, CHD5*, *RPL22* and *TSC2* genes were shared mutation targets in ovarian and colorectal carcinomas. *RPL22* contains a coding repeat which makes it mutation-prone in tumors with MSI [10, 11].

**Figure 3:**
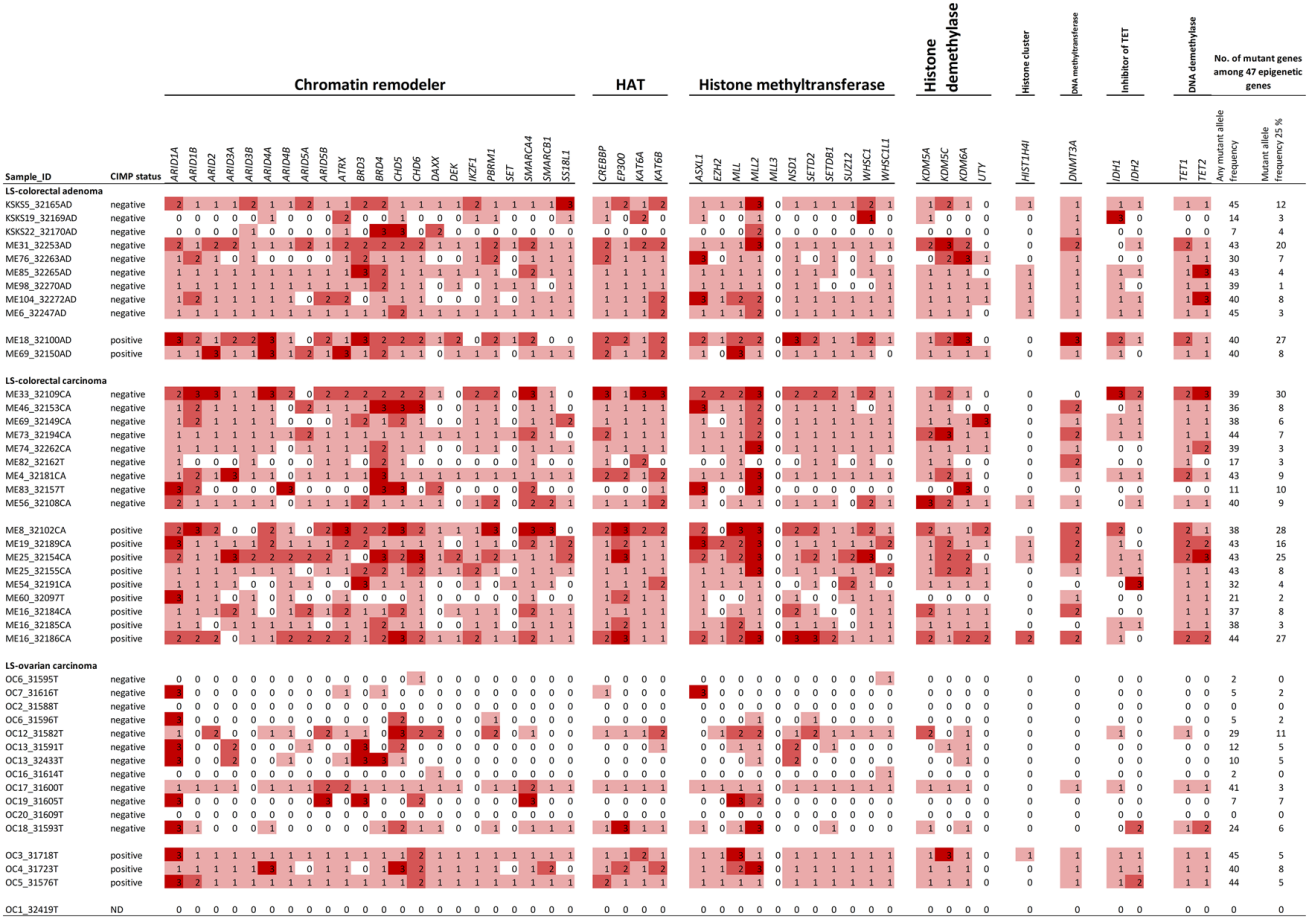
Somatic mutation patterns of all 47 epigenetic key genes included in the Comprehensive Cancer Panel The codes for mutation classification are 0 (white), not mutant; 1 (light red), one or more mutations (truncating or non-truncating) with variant allele frequency < 25 % in tumor tissue; 2 (medium red), one or more non-truncating mutations with variant allele frequency ≥ 25 % in tumor tissue; 3 (dark red), one or more truncating mutations with variant allele frequency ≥ 25 % in tumor tissue.

Genes with a primary function in epigenetic regulation comprised 8 % (47/578) of all genes interrogated in the Comprehensive Cancer Panel. They were significantly enriched among our designated colorectal tumor-associated genes (15/72, 21 %, *p* = 0.0015 by two-tailed Fisher's test) and ovarian carcinoma-associated genes (3/10, 30 %, *p* = 0.045) (Figures 1 and 2). For comparison, DNA repair genes were not at all enriched (these accounted for 4/72, 6 %, of colon tumor-associated genes and 0 % of ovarian tumor-associated genes, whereas the entire panel included 47 such genes, 8 %). The detailed patterns of involvement of all 47 epigenetic key genes examined are shown for each tumor in Figure 3. With a few exceptions, inactivation (loss of function) is expected to underlie pathogenicity of epigenetic regulatory genes [12–14]. A significant fraction of somatic mutations observed in epigenetic regulatory genes in our investigation were truncating frameshift or nonsense mutations compatible with loss of function (Figure 3, Supplementary Table 5, Supplementary Figure 1). In addition, a notable proportion of missense mutations may be pathogenic based on *in silico* predictions (Supplementary Table 5). Some mutations were recurrent, since they had previously been deposited to the COSMIC database (Supplementary Table 5).

## DISCUSSION

DNA MMR deficiency is known to underlie MSI and hypermutability in LS and sporadic tumors, but the mutational landscapes may differ depending on the mechanism of MMR gene inactivation [7, 15]. Even in LS mutation carriers, MSI is not invariably present but shows tumor type-specific variation, likely reflecting different patterns of clonal growth [6]. Of the tumor spectrum in LS, colorectal and ovarian tumors are associated with MSI-H as a rule. The CIMP patterns likewise display tumor type-specific variation in LS, clearly distinguishing gastrointestinal from gynecological tumors [6]. These aspects made us reason that colorectal and ovarian tumors from LS individuals would provide an informative comparative setting to investigate the relationship between somatic mutations and epigenetic aberrations against a shared pattern of MSI-H.

A comprehensive evaluation of the mechanisms of two-hit inactivation identified loss of the wild-type allele as the predominant mechanism of the second hit in both colorectal and ovarian tumors (Table 2). Previous reports are available from LS colorectal tumors [16, 17] with findings that comply with our results. Among tumors without LOH, parallel sequencing detected a somatic point mutation as the likely second hit in most cases. Interestingly, somatic mutations of the predisposing MMR genes were relatively common also in tumors where wild-type allele loss already provided the necessary second hit (Supplementary Table 1). This suggests that multiple clones with different second hits were likely to occur in a single tumor, in agreement with observations of clonal heterogeneity inferred from patterns of microsatellite repeats [18].

The so called neutral evolution with clonal selection occurring before the onset of cancer growth has been proposed as a mechanism of intratumoral heterogeneity [9]. Based on Williams et al. [9], we focused on mutations with allele frequency 25 % or higher to increase the likelihood of clonal (driver) as opposed to subclonal (passenger) mutations. When involvement in at least 31% of tumors was set as an additional requirement, 72 potential driver genes were identified among colorectal tumors (Figure 1) and 10 among ovarian carcinomas (Figure 2). These gene sets had two cardinal features. First, signature genes characteristic of the tumor types in question, such as *APC* in colorectal tumors [1] and *ARID1A*, *PIK3CA*, and *PTEN* in non-serous ovarian carcinomas [19, 20] were well represented. Second, genes responsible for epigenetic regulation were significantly enriched.

*BRD4* encoding a chromatin remodeler and *MLL2* encoding a H3K4 methyltransferase were the top mutant genes in colorectal tumors (62 %) and *ARID1A*, a chromatin remodeler gene, in ovarian carcinomas (50 %). Chromatin remodelers are often affected by inactivating mutations in human cancers, which can result in altered expression of their target genes [21]. However, the occurrence of mutations in *BRD4* specifically has been reported to be low in cancers [22]. Our data revealed high-frequency mutations in *BRD4* or *BRD3* or both (Figure 1) in 79 % (23/29) of colorectal tumors, which is, therefore, a novel finding. Interestingly, BRD4 can act in a tumor-promoting or tumor-protective manner depending on the type of tumors [22]. In colorectal cancer it is mainly tumor-protective [22], which is consistent with our observation of frequent loss-of-function mutations affecting this gene (Figure 3 and Supplementary Table 5). *MLL2* is a known mutational target gene in (MSI) colon cancer [23] and according to recent findings may drive tumorigenesis through genome instability and increased gene mutations [24].

A key finding of our investigation was that the total number of somatic mutations, or mutant genes per tumor, were significantly associated with CIMP. The cause and consequence relationships are unknown. In theory, mutations in genes with epigenetic regulatory function could lead to CIMP. Tahara et al. [25] discovered that somatic mutations in chromatin regulator genes *CHD7* and *CHD8* (neither was included in our targeted gene panel) were enriched in sporadic colorectal carcinomas with CIMP and MSI due to *MLH1* promoter methylation (the CIMP1 subgroup). Genes frequently methylated in CIMP positive tumors were increasingly bound by CHD7 supporting the idea that *CHD7* mutations contributed to CIMP. Conversely, CIMP might induce somatic mutations. Deamination of 5-methylcytosine at CpG dinucleotides is a known mechanism of C to T transitions [26], the predominant SNV type in MSI colorectal cancers [10]. Many repair genes (including *MLH1*) could be affected by promoter methylation as part of CIMP [27], with increased overall mutation frequencies in tumors as a possible consequence. In the study by Tahara et al. [25], CIMP1 colorectal carcinomas had a higher total frequency of somatic mutations compared to CIMP1 colorectal adenomas or non-CIMP colorectal carcinomas (MSS). A possibility remained that the higher mutation rate in CIMP1 colorectal carcinomas primarily reflected their MMR deficiency. In our investigation, MMR status was not a possible confounding factor since it was similar (MSI-H) in CIMP positive and CIMP negative tumors. Contrary to Tahara et al. [25], we saw no difference between colorectal adenomas and carcinomas (Table 3), which was possibly due to the different settings (sporadic and LS, respectively).

Our findings are clinically relevant. Apart from epigenetic regulation, many other central biological pathways (such as DNA repair, Wnt, mTOR, PI3K, NOTCH, MAPK, and tyrosine kinase signaling) are represented among the top mutant genes we identified (Figures 1 and 2, and Supplementary Table 4). Several of these pathways can be therapeutically targeted [28, 29]. A recent study found that *ERBB2*-mutant MSI colorectal cancer was susceptible to irreversible pan-HER inhibitors [30]. The *ERBB2* mutation rate (15 %) in their series fulfilling Bethesda or Amsterdam II criteria was comparable to our LS colorectal tumors with 5/29 (17 %) showing high-frequency mutations. Finally, the general tendency of MMR-deficient tumors to accumulate somatic mutations makes them good candidates for checkpoint blockade-based immunotherapy [31].

Taken together, the causes of CIMP remain elusive and the consequences of this phenomenon are incompletely understood. Our targeted sequencing experiments on LS mutation carriers showed that colorectal and ovarian tumors with CIMP tended to accumulate somatic mutations and mutant cancer-relevant genes, especially those associated with high mutant allele frequencies characteristic of driver genes. Epigenetic regulatory genes were significantly enriched among mutant genes. These findings are interesting and novel and encourage further research. Additional investigations are necessary to address in detail the complex questions regarding the cause and effect relationships. The genes we report mutant are common cancer genes with most listed in the Cancer Gene Census (Supplementary Table 4). Therefore, future research should also be directed to establish the extent to which the associations we found between somatic mutations and CIMP in LS tumors can be recapitulated in the sporadic setting.

## MATERIALS AND METHODS

### Patients and samples

This study included 45 tumor specimens and their corresponding normal samples from 39 LS mutation carriers from the Hereditary Colorectal Cancer Registry of Finland (Table 1). Ovarian carcinomas were all non-serous (13 endometrioid and 3 clear cell) as typical of LS [7]. All tumors and eight normal samples were formalin-fixed paraffin embedded (FFPE) specimens, whereas the remaining normal samples were blood specimens. The average tumor percentage was 46 %, 47 %, and 67 % in colorectal adenoma, colorectal carcinoma and ovarian carcinoma, respectively (Supplementary Table 2). DNA was isolated using non-enzymatic protocols according to Isola et al. [32], and Lahiri and Nurnberger [33] for FFPE and blood specimens, respectively. The Institutional Review Boards of the Helsinki University Central Hospital (466/E6/01) and Central Finland Health Care District (10U/2011) approved this study. The National Supervisory Authority for Welfare and Health (Dnro 1272/04/044/07 and Dnro 10741/06.01.03.01/2015) approved the collection of archival specimens.

### Microsatellite instability (MSI) analysis

MSI status was determined using mononucleotide repeat markers *BAT25* and *BAT26* that are specific and sensitive indicators of high-degree MSI (MSI-H) [34, 35].

### CpG island methylator phenotype (CIMP) status

Colorectal tumors were investigated by methylation-specific multiplex ligation-dependent probe amplification (MS-MLPA) using SALSA MS-MLPA probemix ME042-B2 (MRC Holland, Amsterdam, The Netherlands (http://mrc-holland.com) as described [8]). Samples were classified CIMP positive when at least 3 of 5 genes from the Weisenberger et al. [36] panel (*CACNA1G, IGF2, NEUROG1, RUNX3*, and *SOCS1)* were methylated. For ovariantumors, no established CIMP criteria exist, and the corresponding methylator phenotype was determined using SALSA MS-MLPA probemix ME001-C1 (MRC Holland, Amsterdam, The Netherlands) for 24 tumor suppressor genes as described [7]. According to our previous experience [37], a tumor was considered CIMP positive when 5 or more genes were methylated.

### Panel sequencing

Sequencing was conducted at the Institute for Molecular Medicine Finland (FIMM; Helsinki, Finland) using the Nimblegen Comprehensive Cancer Panel (Roche Diagnostics), a 4Mb design with 578 cancer-related genes compiled from the Sanger Institute Cancer Gene Census Database and the NCBI Gene tests databases. Libraries were prepared using ThruPLEX^®^ DNA-seq Kit, and the exons captured according to the manufacturer's protocol (Rubicon Genomics). Sequencing was performed on Illumina HiSeq 2500 platform (San Diego, CA). The mean target coverage was 41-fold for colorectal and 72-fold for ovarian tumors (Supplementary Table 6).

The pipeline used for variant calling is described in Sulonen et al. [38] Raw Illumina reads were first merged with SeqPrep. Resulting paired reads were trimmed of B blocks in the quality scores from the end of the read. Trimmed reads shorter than 36 base pairs were removed. Reads were aligned using the Burrows-Wheeler Aligner version 0.6.2 [39] against the human genome GRCh37 reference-genome primary assembly. Reads mapping to multiple genomic positions were removed. The alignment was refined using GATK Indel Realignment version 3.4. After the alignment, potential PCR duplicates were removed with Picard MarkDuplicates version 1.90.

### Somatic mutation analysis

Non-synonymous somatic mutations (missense, nonsense, frameshift, in-frame coding deletion/insertion and splice site mutations) were identified from the paired normal and tumor data using the VarScan 2 mutation detection algorithm version 2.3.2 [40]. The following parameters were used for calling high-confidence somatic mutations: strand-filter 1, min-coverage-normal 8, min-coverage-tumor 6, somatic-*p*-value 1, normal-purity 1, and min-var-freq 0.05. Mutations were annotated using SnpEff version 4.0 [41] with the Ensembl v68 annotation database [42]. To filter out misclassified germline variants, the common population variants included in the Database of Single Nucleotide Polymorphisms (dbSNP; https://www.ncbi.nlm.nih.gov/snp) were removed. Variants with VarScan somatic *p*-value below 0.01 were selected for subsequent analyses.

Throughout this paper, the term “mutation” is used for any non-synonymous sequence change with the possibility of being pathogenic (including traditional pathogenicity classes 3 -5).

### Second hit analysis of DNA mismatch repair (MMR) genes

LOH analyses took advantage of the predisposing mutation. When the predisposing mutation was a point mutation, LOH analysis was performed utilizing VCP filtered sequencing data (.vcf-files) on the predisposing MMR gene mutation regions obtained from tumor and normal samples by VarSeq (GoldenHelix^®^). The ratio of variant allele (Alt) to reference allele (Ref) reads was determined in tumor (T) and matching normal (N) DNA and the LOH ratio calculated using the following formula: R = (Alt:Ref)_T_/(Alt:Ref)_N_. The thresholds for LOH and putative LOH are specified in Ollikainen et al. [43] When the predisposing MMR mutation was a large deletion, LOH analysis was performed by MLPA (with SALSA P003-C1 for *MLH1* and *MSH2* and 072-C1 for *MSH6*, MRC Holland, Amsterdam, The Netherlands), and the results interpreted according to Zhang et al. [44].

Data on somatic point mutations in *MLH1*, *MSH2*, and *MSH6* were obtained as part of the Nimblegen Comprehensive Cancer Panel. The promoters of *MLH1*, *MSH2*, and *MSH6* were investigated for methylation by MS-MLPA as described in Valo et al. [8] and Niskakoski et al. [7].

### In silico evaluation of somatic mutations for pathogenicity

*In silico* evaluation of somatic single nucleotide variants (SNVs) was conducted using VarSeq (GoldenHelix^®^) (Supplementary Tables 1 and 5). VarSeq includes 6 individual algorithms to predict the effect of amino acid substitution on protein function: SIFT (http://sift.jcvi.org/), PolyPhen-2 [45], MutationTaster [46], MutationAssessor (http://mutationassessor.org/r3/), FATHMM [47–49], and FATHMM MKL Coding (http://fathmm.biocompute.org.uk/). In the second hit analysis (Supplementary Table 1), splicing consequences of SNVs in splice site regions were predicted using Human Splicing Finder (http://www.umd.be/HSF3/). Somatic mutations in MMR genes were checked against the InSIGHT database (Leiden Open Variation Database, LOVD v. 2.0 Build 36; http://chromium.lovd.nl/LOVD2/colon_cancer/home.php) for pathogenicity classifications (Supplementary Table 1). Somatic mutations in MMR genes as well as those affecting the top 72 colorectal tumor and 10 ovarian tumor-associated genes were also assessed for possible presence in the Catalogue of somatic mutations in cancer (COSMIC v71, GRCh 37; http://grch37-cancer.sanger.ac.uk/cosmic) (Supplementary Tables 1 and 5).

### Statistical analyses

Statistical analyses were conducted using the SPSS software, version 24.0 (IBM SPSS Inc., Chicago, IL, USA). The data were first checked for the applicability of parametric vs. non-parametric tests. Differences between the distributions of mutations or mutant genes in two independent groups were evaluated for statistical significance by the Mann-Whitney U test. The Fisher's exact test was used for pairwise comparisons of frequency data. Correlations were assessed by determining the Pearson correlation coefficient for parametric data. Two-tailed *p*-values < 0.05 were considered significant.

## SUPPLEMENTARY MATERIALS FIGURES AND TABLES















## Abbreviations

LS: Lynch syndrome
CIMP: CpG island methylator phenotype
MSI: microsatellite instability
MSI-H: high-degree MSI
MMR: mismatch repair
LOH: loss of heterozygosity
FFPE: formalin-fixed paraffin embedded
MS-MLPA: methylation-specific multiplex ligation-dependent probe amplification
Alt: variant allele
Ref: reference allele
SNV: single nucleotide variant.
