# Supplementary material for: Sequencing of Lynch syndrome tumors reveals the importance of epigenetic alterations
Source: Oncotarget. 2017 Nov 14;8(64):108020–30. doi: 10.18632/oncotarget.22445 (PMC5746122; doi:10.18632/oncotarget.22445)
Supplement: Supplementary file 1 [file oncotarget-08-108020-s001.pdf]

Sequencing of Lynch syndrome tumors reveals the importance of epigenetic alterations

SUPPLEMENTARY MATERIALS

Relative shares of somatic mutations stratified by mutant allele frequency and mutation type

A. Epigenetic genes in colorectal specimens (n = 29)

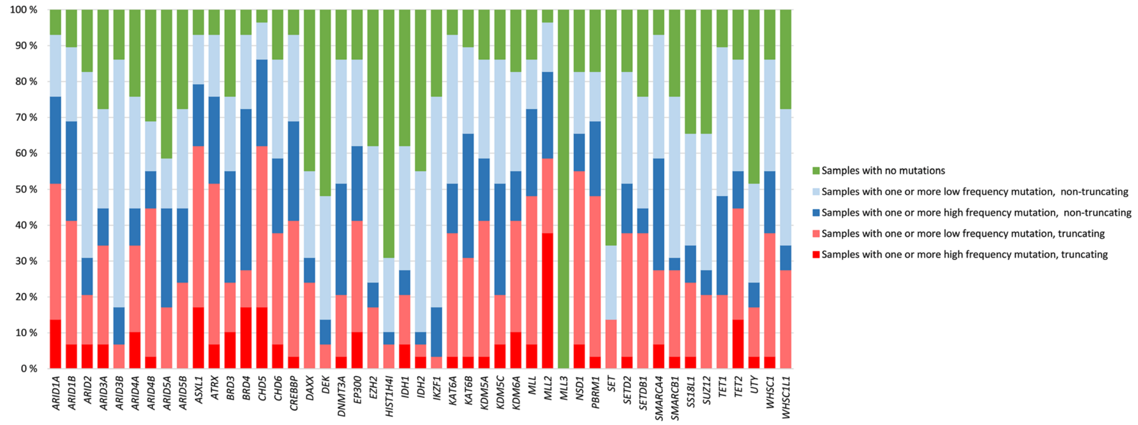

B. Epigenetic genes in ovarian carcinomas (n = 16)

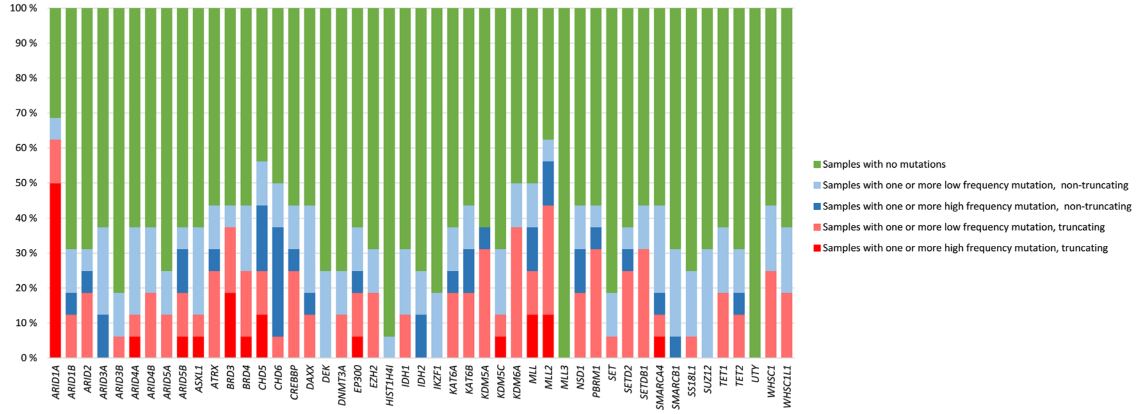

(*Continued*)

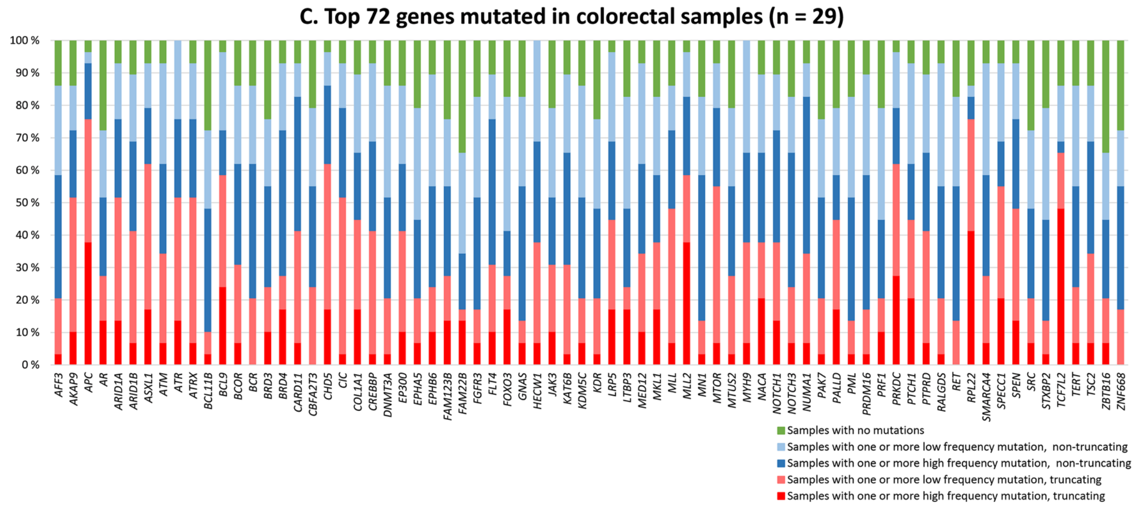

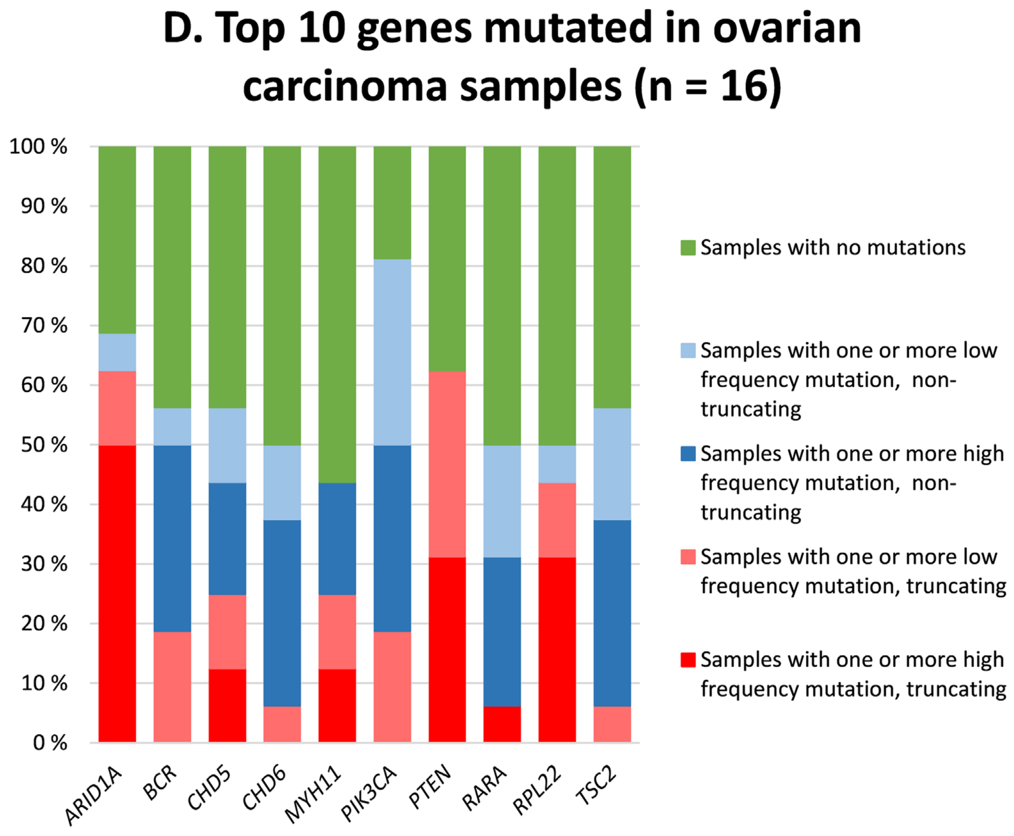

Supplementary Figure 1: (A-D) Relative shares of somatic mutations stratified by mutant allele frequency and mutation type.

Supplementary Table 1: Somatic point mutations as second hits in LS tumors stratified by LOH

See Supplementary File 1

Supplementary Table 2: Correlation between promoter methylation and somatic mutation tendencies

See Supplementary File 2

Supplementary Table 3: Distributions of genes according to their tendency to acquire high-frequency mutations

See Supplementary File 3

Supplementary Table 4: Pathway annotations of top 72 colorectal tumor and top 10 ovarian carcinoma-associated genes

See Supplementary File 4

Supplementary Table 5: Specification of all somatic mutations detected in 72 colorectal tumor and 10 ovarian carcinoma-associated genes

See Supplementary File 5

Supplementary Table 6: Performance characteristics of panel sequencing

See Supplememtary File 6
